# Who Are the Managers?

**Published:** 1873-04-01

**Authors:** 


### Medical Examiner.

*A Semi-Monthly Journal of Medical Sciences.*

EDITED BY

N. S. DAVIS, M. D., AND F. H. DAVIS, M. D.

Chicago, April 1st, 1873.

### EDITORIAL.

### WHO ARE THE MANAGERS?

The *Medical Record* recently closed a brief notice of the Transactions of the American Medical Association as follows: "We would make this suggestion to the *managers* of the American Medical Association: *First*, Let the transactions be confined to the reports of committees, abridged by the committee of publication, the lists of members, minutes of the meetings, and other miscellaneous matter. A volume thus constituted would be small and compact, and valuable for reference. *Secondly*, Let the papers read in the Sections —if they have any value—be recommended for publication in some medical journal." We do not know who the editor of the *Record* means by *managers* of the Association. We find no officers, or committee, or board, with that title, provided for in the Constitution or By-Laws; and we doubt whether it is either proper or just to make use of such phraseology as implies that the Association is under the dictation or control of a few individuals or "managers." It is calculated to impose an unjust responsibility upon a few; and, in the same ratio, to deter members generally from freely exercising their right of perfect equality, both in originating and discussing all matters pertaining to the interests of the Association or of the profession which it represents. Neither can we fully approve of either of the writer's suggestions. A volume of transactions, containing only the lists of members, the record of proceedings, and abridgements of the reports of committees, would not be worth the paper on which it would be printed; simply because the *Medical Record*, and every other enterprising medical journal in the country, would have substantially the same matter in their columns, and before the profession, weeks before the committee of publication could get it through the press in official form. We believe the volume of transactions should contain, in addition to the record of proceedings and the official reports of standing committees, all papers read in the Sections, embodying original investigations of value in any of the departments of medical science and art. All other papers should be either recommended for publication in some medical periodical, or returned to their authors, as their merits might indicate. And each Section, in order to make the discrimination necessary, should refer every paper presented to it to a sub-committee for detailed examination and final report to the permanent Secretary of the Association, within thirty days after the adjournment of the annual meeting. And precisely this mode of sifting and classifying the reports and papers presented in the several Sections, was provided for by those whom the *Record* is pleased to call "managers" of the Association eight or ten years since. On page 674 of the volume of transactions for 1872, at the head of the list of ordinances now in force, are the following resolutions, adopted expressly for the guidance of the Sections at the meeting in Boston, in May, 1865:

*Resolved*, That the several Sections of this Association be requested, in the future, to refer no papers or reports to the Committee of Publication, except such as can be fairly classed under one of the three following heads, viz.: *First*, Such as may contain and establish *positively* new facts, modes of practice, or principles of real value. *Second*, Such as may contain the results of well-devised original experimental researches. *Third*, Such as present so complete a review of the facts on any particular subject as to enable the writer to deduce therefrom legitimate conclusions of importance.

*Resolved*, That the several Sections be requested, in the future, to refer all such papers as may be presented to them for examination by this Association, that contain matter of more of less value, and yet cannot be fairly ranked under either of the heads mentioned in the foregoing resolutions, back to their authors, with the recommendation that they be published in such regular medical periodicals as said authors may select, with the privilege of placing at the head of such papers, "Read to the —— Section of the American Medical Association, on the —— day of ——, 18—."—(*Vide* Transactions, vol. xvi, p. 40.)

If these directions were faithfully observed by each *Section*, the resulting volume of Transactions for each year, would be "small and compact" enough to satisfy even the *Medical Record.*

